# Taxonomic remarks about *Semiclivina* (Kult, 1947) new status, with description of *Uroclivina* subgen. n., and of two new species from South America (Coleoptera, Carabidae, Scaritinae, Clivinini)

**DOI:** 10.3897/zookeys.132.1508

**Published:** 2011-10-03

**Authors:** Alexander Dostal

**Affiliations:** 1 Duchekgasse 39, Vienna, Austria

**Keywords:** Coleoptera, Carabidae, Scaritinae, Clivinini, *Semiclivina*, *Uroclivina*, South America, Argentina, French Guyana, identification key

## Abstract

The subgenus *Semiclivina* Kult, 1947 of *Clivina* Latreille, 1802 (*sensu lato*) has been re-ranked as a genus, with the most readily observed feature being the stridulation organ of the proepisterna and front femora. A group of species within *Semiclivina* is characterized by a peculiar acute tubercle at the posterior margin of the eye, which corresponds to an equally noticeable incision of the anterior margin of the pronotum. This group is considered as monophyletic and placed as such in the **subgen. n.** *Uroclivina*. The species *Semiclivina (Uroclivina) bergeri* **sp. n.** from Argentina and southern Brazil and *Semiclivina (Uroclivina) schmidi* **sp. n.** from French Guyana are described. The following additional species are included in *Uroclivina*: *Clivina urophthalmoides* (Kult, 1947) new combination, *Clivina urophthalma* (Putzeys, 1863) new combination, and *Clivina oxyomma* (Putzeys, 1868) new combination. The two subgenera of *Semiclivina* Kult, and the current five species of *Uroclivina* are differentiated in a key.

## Abstract (de)

Die Untergattung *Semiclivina* Kult, 1947 der Großgattung *Clivina* Latreille, 1802 ist durch das Stridulationsorgan an den Proepisternen besonders auffällig und wird als eigenständige Gattung etabliert. Innerhalb dieser Gattung ist eine Artengruppe durch ein Tuberkel am hinteren Augenrand und der korrespondierenden Einkerbung am Vorderrand des Pronotums besonders auffällig. Diese Artengruppe wird als monophyletische Gruppe angesehen und als solche in der Untergattung *Uroclivina* nov. zusammengefasst. Die Arten *Semiclivina (Uroclivina) bergeri* **sp. n.** aus Argentinien und S-Brasilien sowie *Semiclivina (Uroclivina) schmidi* **sp. n.** aus französisch Guyana werden beschrieben. Folgende weitere Arten werden in die neue Untergattung gestellt: *Clivina urophthalmoides* (Kult, 1947) **comb. n.**, *Clivina urophthalma* (Putzeys, 1863) **comb. n.** und *Clivina oxyomma* (Putzeys, 1868) **comb. n.** In einer Bestimmungstabelle werden die beiden Untergattungen von *Semiclivina* Kult, 1947, sowie die fünf Arten der Untergattung *Uroclivina* nov. aufgeschlüsselt.

## Introduction

Currently, the genus *Clivina* Latreille, 1802 includes 456 taxa (species and subspecies) which are arranged worldwide in 12 subgenera: *Antroforceps* (Barr, 1967); *Clivina* s. str.; *Cliviniana* Kult, 1959; *Cliviniella* Kult, 1959; *Dacca* (Putzeys, 1861), *Eoclivina* Kult, 1959; *Isoclivina* Kult, 1959; *Leucocara* Bousquet, 2009; *Paraclivina* Kult, 1947; *Physoclivina* Kult, 1959; *Reichardtula* Whitehead, 1977; *Semiclivina* Kult, 1947). Some of these subgenera are well defined by certain characters and probably to be considered as proper genera in future. One of these is *Semiclivina* Kult, 1947, which is ranked as a genus below. The availability of numerous specimens of this genus led to the investigation of a well characterized species group, which is treated here as an independent subgenus.

## Material and methods

Preserved specimens from different collections are used which are mounted on commercially available paper cards. I strongly recommend cutting away the right upper corner of the mounting card, for more convenient investigation of the lower surface of the pronotum, and to remove the abdomen and to mount this, ventral side up, beside the specimen, because the abdomen carries important distinguishing characters. Male and female genital organs were dissected and also glued to the cards beneath the specimens from which they were removed.

The species descriptions were based on the most distinguishing external characters as defined by Baehr (2008: 9). Label data for examined material are given in full, with exact labeling, except for the date format, which is transcribed to the format “dd.mm.jjjj”.

Abbreviations of collections mentioned in text:

CBP	Collection Petr Bulirsch, Praha

CBM	Collection Martin Baehr, München

CDW	Collection Alexander Dostal Wien, including the collection Karel Kult

IRSNB	Institut Royal des Sciences Naturelles, Bruxelles

MNHP	Museum National d’Histoire Naturelle, Paris

ZMHB	Museum für Naturkunde der Humboldt Universität Berlin

NMW	Naturhistorisches Museum Wien

### Measurements

Measurements were taken with a calibrated Leica ocular scale at absolute magnifications 19,4× (for all measurements except pronotum) and 39,1× (for pronotal length and width). L = total length in mm, from Apex of Mandible to apex of elytra. W = maximum width in mm, situated in the apical third of elytra. PL = pronotum length, maximum length of pronotum measured along median line from the base of the anterior bristle fringe to the base of the posterior one. PW = maximum width of pronotum, measured normal to the midline, situated in most cases near the posterior angles P-LW = length-width -index of pronotum (length:width), if the value is smaller than 1, it means that the pronotum is wider than long, for values above 1: the pronotum is longer than wide. F-LW = length-width - index of both elytra, same as previous. Dl, Dr = number of dorsal setiferous punctures (D) in the third interval of the left side (Dl) and of the right side (Dr) respectively. The preapical puncture in the third interval is counted together with the other discal punctures.

### Statistics

Following parameter are calculated: M = arithmetical mean, Max = maximum value, Min = minimum value, N = number of individuals measured, SD = standard deviation.

#### Genus Semiclivina

Kult, 1947 stat. n.

http://species-id.net/wiki/Semiclivina

Clivina  Subgenus *Semiclivina*Kult 1947: 31–32; Reichardt 1977: 391; Nichols 1988a: 154; 1988b: 91; Ball 2001: 136; Lorenz 2005: 145; Baehr 2008: 23; Bousquet 2009: 41.

##### Type Species.

*Clivina dentipes* Dejaen, 1825, by original designation: Kult 1947: 31.

**Figure 1.**
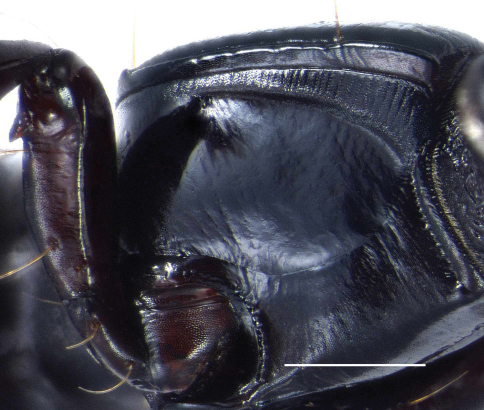
Photograph of prothorax and base of metathorax of *Semiclivina* species; right lateral view, showing the stridulation ridge (arrow); Scale bar: 0,5 mm.

**Figure 2.**
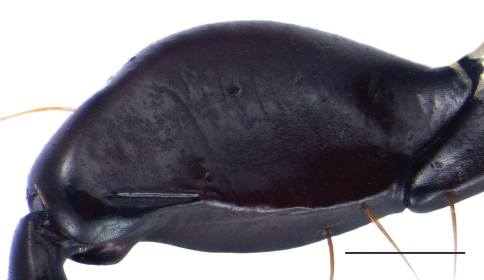
Photograph of right leg, posterior view, of base of trochanter, femur, and base of tibia of *Semiclivina* species, showing stridulation ridge on inner side of profemur (arrow); Scale bar: 0,5 mm.

The genus *Semiclivina* (Kult, 1947) is readily recognized by the sculptured band of the proepisternum, extended more or less parallel to the proepisternal margin in the basal part of the proepisternum, curved inward in the anterior part, ended at the anterior end of the proepisternal-prosternal suture (Fig. 1). This structure was differently interpreted by diverse authors (Bousquet 2009: 38) as furrow, ridge or carina or elongate striole (Baehr 2008: 23). In fact it is a slightly elevated band like structure consisting of very fine, regular transverse ridges. This sculptured band (Bousquet 2009: 38) is part of a stridulation organ in the sense of a pars stridens, whereas the corresponding plectrum is a fine, sharp longitudinal ridge, just above the distal part of the lower inner edge of the profemur (Fig. 2). In some species the outer edge of the profemur is produced to a remarkable denticle, but this structure does not interact with the stridulation organ in any way. The function of this stridulation organ has not been observed so far.

This organ is not unique within the Tribe Clivinini: exactly the same structures (the stridulation ridge on proepisterna and the ridge on the distal part of the inner edge of the profemur) together with other features separates the ardistomine genus *Ardistomis* Putzeys, 1846 from *Semiardistomis* Kult, 1950, but it is unique within *Clivina* (sensu lato), forming a very isolated, distinct group, restricted to the western hemisphere and to a single species from Australia (New South Wales) described by Baehr 2008: 23–25. Within the tribe Clivinini no other genera than *Ardistomis* Putzeys, 1846 and *Semiclivina* (Kult, 1947) carry a stridulation organ on the proepisternum. Beside this, *Semiclivina* is characterized by a distal spine-like projection at the outer end of the mesotibia, which is small in some taxa, but clearly identifiable by the apical seta excentrically inserted, in contradiction to a tubercle, which carries the seta at the top of the tubercle (see also Ball 2001: 136, 140; Bousquet 2009: 41, 43); usually five setae in elytral interval 3 (including preapical puncture); elytral striae 1 to 5 free at base (between humeral channel and suture, the basal keel of some species and the flat tubercles at the end of intervals 2 to 5 do not limit this definition); profemur with a sometimes small dentiform projection at the outer edge toward apex; anal ventrite (abdominal sternum VII) with two marginal punctures at each side relatively close together. The species included in *Semiclivina* are listed in Bousquet (2009: 39–41), where the assignment of some species remains uncertain. It is most likely, that species not included in the subgen. n. *Uroclivina* comprise a more or less heterogeneous group which form a probably paraphyletic group, defined as *Semiclivina* (s. str.). This subgenus will be the objective of further investigations in the future.

#### Subgenus Uroclivina subgen. n.

urn:lsid:zoobank.org:act:581B1ABC-14ED-4D8B-B216-7119CF90DCDC

http://species-id.net/wiki/Uroclivina

##### Type species.

*Semiclivina bergeri* sp. n., herewith by original designation; it is the most abundant species of this subgenus.

##### Etymology.

combination from the genus-name “*Clivina*” and the specific epithet “*urophthalma*”.

##### Recognition.

the species of this subgenus are characterized by the denticle of the posterior margin of the eye, the corresponding incision in the anterior margin of the pronotum at anterior angle and the sculptured band (stridulation organ) on the proepisterna, the latter is the most obvious character of the genus *Semiclivina*.

##### Description.

**Figure 3.**
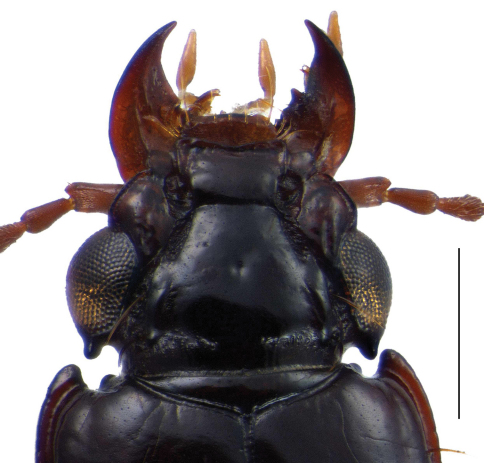
Photograph of head and anterior portion of pronotum, dorsal view, of *Semiclivina bergeri* new species, Holotype, showing **a** tubercle at posterior margin of eye **b** incision of anterior margin of pronotum; Scale bar: 0,5 mm.

*Head*: clypeus middle part slightly produced, separated from wings, anterior margin slightly concave, clypeus posteriorly separated from frons by a transverse furrow; posterior margin of eye with an acute dentiform tubercle (Fig. 3a); antennal scape distally with a seta, antennomeres 1 and 2 without pubescence, antennomeres 3 to 11 densely pubescent and longer than wide; labrum 7-setose; penultimate palpomere of labial palpus bisetose. *Pronotum*: with a marginal seta at the end of the anterior fifth and a second one at hind angle; basal border fine, just above the peduncle, finely bordered between basis and hind angle, and from hind angle to markedly produced anterior angles, border in the anterior third broader, anterior margin with a sharp, narrow incision just beneath the front angles, corresponding with the tubercle at hind margin of eye (Fig. 3b); middle furrow and front transverse furrow clearly impressed; proepisterna with a stridulation band. *Elytra*: scutellar puncture present, scutellar striole indistinct, marked as short furrow just beneath the suture, or not evident; elytral striae 1 to 5 free at base, means within the humeral denticle and suture; basis at the end of interval 1 to 5 sometimes with flat tubercles, which may join with transverse basal keel; elytral intervals distinctly punctured; interval 3 with 5 setiferous punctures. *Legs*: front femur with a sharp longitudinal stridulation ridge, just above the distal part of the lower inner edge of the profemur; mesotibia with a small distal spur. *Abdomen*: abdominal sterna IV to VI with a paramedian seta on each side, sterna V to VII with a basal transversal ridge; abdominal sternum VII with a pair of marginal setae on each side relatively close together, all abdominal sterna microsculptured.

##### Geographical distribution.

The species are restricted to South America, ranging from French Guyana, southward through the Amazon Basin to southern Brazil, Paraguay, North and Central Argentina and most likely also Uruguay (no evidence from this country so far).

**Way of life**. most specimens of *Clivina* (*sensu lato*) usually live near wet places or in swamps. Some species, like *Clivina fossor* (Linnaeus, 1758) are not bound to humid habitats. Most of the species are macropterous, capable of flight mostly during night, especially at temperatures above 25°C. Specimens of *Uroclivina* obviously live also close to humid habitats: they were collected at light and sometimes in extraordinary large series near rivers, lakes or other wet places.

##### Key to subgenera of *Semiclivina* and species of the Subgenus *Uroclivina*

**Table:** 

1	Posterior part of eye rounded, without denticle; pronotum without incision of the anterior margin	Subgenus *Semiclivina* (s. str.) Kult, 1947
–	Posterior part of eye extended to a sharp denticle; pronotum (Fig. 3) with a sharp incision of the anterior margin *Uroclivina*, subgen. n.	2
2	Elytral base unbordered and without tubercles, except one beside the scutellar puncture	3
–	Elytral base bordered or with tubercles at the basal end of intervals 2, 3, 4 and 5	4
3	Abdominal sterna, especially sternum VII (anal ventrite) with gross isodiametric microsculpture, all without punctures; median lobe of aedeagus slender, rounded at apex, not markedly flattened laterally (Fig. 7 a,b); distal spur at the outer edge of mesotibia short, about 1.5× as long as wide; punctuation of neck fine, punctures larger in the middle, nearly uninterrupted at middle; on average smaller species, length 4.2 mm; Brazil: Mato Grosso, Corumba	*Semiclivina (Uroclivina) urophthalmoides* (Kult, 1947)
–	Abdominal sterna with fine isodiametric microsculpture, without punctures (Fig. 4a); median lobe of aedeagus flattened at apex to a sharp edge (Fig. 6 a,b); distal spur at the outer edge of mesotibia longer, more than 2× as long as wide; punctuation of neck very fine, interrupted at middle; on average larger species, length 5.2–5.7 mm; Argentina, Brazil: Mato Grosso; one of the most abundant species in this area.	*Semiclivina (Uroclivina) bergeri* spec. nov. (= *marquardti* Van Emden, (museum label name), Argentina: Santa Fe)
4	Base of elytra not continuously bordered, but with flat tubercles at the basal end of intervals 3, 4, and 5, and a tubercle beside the scutellar puncture; anal ventrite with fine, slightly transverse mashes, without punctures (Fig. 4d); pronotum slightly longer than wide (1.04×); smaller and slender species, body cylindriform, inner elytral intervals slightly convex; length 5.6 mm;	*Semiclivina (Uroclivina) urophthalma* (Putzeys, 1863)
–	Base of elytra bordered at least at the basal end of interval 4 and 5 and with a more or less confluent tubercle at the end of interval 3, and a tubercle beside the scutellar puncture; anal ventrite at least with fine punctures; body flattened, inner elytral intervals flat; species longer than 6 mm	5
5	Larger species, length 7.6 mm; pronotum as wide as long (1.05×); anal ventrite with slightly transverse mashes and an area with about 40–50 more gross punctures at each side (Fig. 4c); Brazil: Bahia	*Semiclivina (Uroclivina) oxyomma* (Putzeys, 1868)
–	Smaller species, length 6.17–6.43 mm; anal ventrite with extremely fine, slightly transverse mashes and an area with about 10 to 15 fine and shallow punctures at each side(Fig. 4b); French Guyana	*Semiclivina (Uroclivina) schmidi* sp. n.

#### Semiclivina (Uroclivina) bergeri sp. n.

urn:lsid:zoobank.org:act:C60E1149-8355-456A-BDB5-62634D6B028D

http://species-id.net/wiki/Semiclivina_(Uroclivina)_bergeri

Fig. 3 4a 5 6

Clivina (Semiclivina) marquardti Van Emden 1949: 861–863, museum label name

##### Specific epithet.

Latinized noun, genitive case, an eponym based on the surname of my friend and partner, Helmut Berger jun., who organizes and joins me in my entomological excursions all over the world.

##### Recognition.

A typical *Uroclivina* – species with basal border of elytra without a keel, elytral stria 1 in the basal part closely joining suture; abdominal sterna without punctuation, but with distinct microsculpture, median lobe of aedeagus flattened, blade-like.

##### Description.

**Figure 4.**
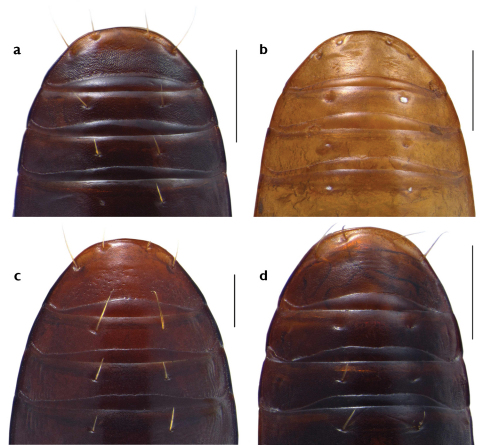
Photograph of abdominal sterna IV-VII, ventral view, of **a** *Semiclivina bergeri*, sp. n., Holotype **b** *Semiclivina schmidi* sp. n., Holotype **c** *Semiclivina oxyomma* (Putzeys) **d** *Semiclivina urophthalma*(Putzeys); Scale bars: 0,5 mm.

*Color*: mature individuals are unicolorous dark brown to piceous, with annexes lighter, reddish-brown, immature ones are all variations lighter up to light yellow-brown, usually all stages are met within a population. *Microsculpture*: glossy, pronotum and elytra with a fine, sometimes barely visible, more or less isodiametric microsculpture, but still glossy *Head* (Fig. 3): middle part of clypeus more produced than lateral wings, anterior margin slightly concave, bordered, wings unbordered; preocular area finely bordered; upper surface of clypeus glossy without microsculpture, except microscopic fine punctures; clypeus divided from frons by a transverse furrow; longitudinal sulci broad, ground microreticulate, bearing a seta at basal level of clypeus on each side; frons glossy, with a very shallow central foveola; above supraorbital setae with a broad ridge, medially bordering a small longitudinal reticulate groove; neck finely punctuate, interrupted at middle; posterior margin of eye with the characteristic tubercle. *Pronotum*: convex, disc somewhat flattened, about as wide as long (range 0,88x to 1,04x, see Table 1); surface glossy, but with fine microreticulation, fine transverse wrinkles in the basal two thirds, declivity at base with transverse wrinkles more dense and distinctly shagreened; anterior angles strongly produced forward, mediad with a sharp and narrow incision, which corresponds to the postorbital tubercle. *Elytra*: about twice long as wide (range: 1.93× – 2.11×, see Table 1); subparallel, convex at sides, disc somewhat flattened, glossy, with very fine microreticulation; elytral striae distinctly engraved from base to apex, with gross punctures at base which become finer towards apex; 7^th^ and 8^th^ interval narrowed at base and sometimes elevated to a keel; first elytral stria bending inwards towards suture at base, from the second sixth onwards it continues parallel to suture up to the apex. *Legs*: fore-tibia 4 dentate, the proximal one very small, triangular, upper surface distinctly sulcate; mesotibia with a small distal spur on the upper edge, which is about two times longer than wide. *Abdominal sterna* (Fig. 4a): IV to VI finely transversally microreticulate, isodiametric at sides, VII completely isodiametrically microsculptured, with 2 pairs of marginal setae in both sexes, relatively close together. *Male genitalia* (Fig. 6a-d): median lobe blade-like flattened, apex spatulate; parameres long and slender, left one about twice as broad as the right one, with 2 setae at apex, right one with an apical fringe of about 10 setae. *Female genitalia*: stylus tall, slender, bent inwards, apex acute, lateral with one big seta, ventral with 4 smaller setae (Fig. 6e-f).

**Table 1.** Descriptive statistics for *Semiclivina* (*Uroclivina*) bergeri spec. nov. from the population sample south of Corrientes, Argentina (N = 114).

	**P-LW**	**E-LW**	**L (mm)**	**W (mm)**	**PL (mm)**	**PW (mm)**	**Dl**	**Dr**
**Min**	0.88	1.93	4.49	1.22	1.07	1.11	5.00	5.00
**Max**	1.04	2.11	6.32	1.68	1.53	1.59	5.00	5.00
**Mean**	0.97	2.01	5.41	1.44	1.28	1.33	5.00	5.00
**SD**	0.03	0.04	0.35	0.09	0.09	0.09	0.00	0.00
**Holotype**	0.95	2.04	5.25	1.38	1.22	1.29	5	5

**Figure 5.**
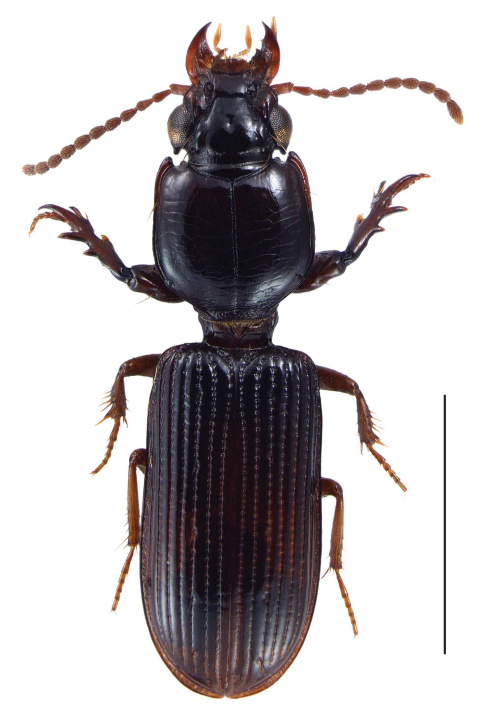
Photograph of habitus of holotype of *Semiclivina bergeri*sp. n., dorsal view; Scale bar: 2 mm.

**Figure 6.**
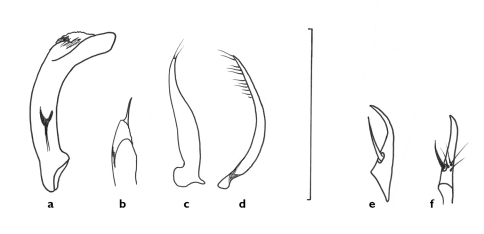
Line drawings of genitalia of *Semiclivina bergeri* sp. n. **a-d** holotype- **a** median lobe, left lateral view **b** median lobe, apical portion, dorsal aspect **c** and **d** left and right paramere, respectively, ventral view **e-f** paratype, ovipositor left stylomere **e** lateral aspect **f** medial aspect. Scale bar = 1 mm.

##### Measurements.

see Table 1; length 4.49–6.32 mm, width 1.22–1.68 mm

**Geographical distribution**. The range of this species extends from the Mato Grosso of Brazil southward to Paraguay and Argentina.

##### Material examined.

*Holotype*: ♂, Argentina NE, S of Corrientes, River Parana, 16. 01. 2009, leg. M.

Snizek, (CDW). 4247 *Paratypes*: **Argentina:** 2 Ex., Argentine Republic, Villa Ana, F.C.S. Fe, December 1924, K.J. Hayward, Paratype Clivina marquardti Van Emden, (CDW); 1 Ex., dtto, January 1926, (CDW); 1 ♀, dtto, December 1925, at light, (CDW); 1 ♂, Argentine, Prov. Corrientes, zw. Lago Ibera & Santo Tome, 26. 09. 1997, (CDW); 84 ♂, 69 ♀, 4046 Ex, Argentina NE, S of Corrientes, River Parana, 16. 01. 2009, leg. M. Snizek, (CBP, CDW, CBM, NMW); 1 ♂, 1 ♀, Argentina, NC, Gran Chaco, Salada riv., S of Macapilo (SE Salta), 20.01.2009, leg. M. Snizek, (CDW); 3 ♂, 3 ♀, Argentina NW, Salta prov., Chicoana riv., El Carril, 28.01.2009, leg. M. Snizek, (CDW); 6 ♂, 8 ♀, Argentina NW, Salta prov., Andes mts., N of Cachi, 2600 m, 25. 01. 2009, leg. M. Snizek, (CDW); 8 ♂, 5 ♀, Argentina N, S of Salta (50 km), E of Coronel Moldes, 23. 01. 2009, leg. M. Snizek, (CDW); 1 Ex., S-Amerika: Argentinia, Prov. Entre Rios/Dept Colon, 5.-10.II.1989, leg. Liebig, (CBM); **Brazil:** 2 ♂, 1 ♀, Corumba, Matt. Grosso, Cl. urophthalmoides Kult, Paratypes (CDW); 3 ♂, Corumba, Matt. Grosso, Cl. urophthalmoides Kult, (CDW); **Paraguay:** 2 Ex. Paraguay, S. Antonio, (CDW); 1 ♂ Paraguay, Prov. Pres Hayes, Buffalo Bill, 23.16S 58.54W 108 m, 01.12.2010 Sv. Bily leg., (CDW); 2 Ex., Paraguay Asuncion, 2.X.1991, (CBM).

#### Semiclivina (Uroclivina) schmidi sp. n.

urn:lsid:zoobank.org:act:2BB78AC8-BC09-4F3C-885F-1894BF971101

http://species-id.net/wiki/Semiclivina_(Uroclivina)_schmidi

Fig. 4b 8 9 

##### Specific epithet.

Latinized noun, genitive case, an eponym based on the surname of the collector of this species, my colleague and friend Herbert Schmid.

##### Recognition.

a typical *Uroclivina* – species with base of elytra with a keel at the end of intervals 3 to 5; elytral stria 1 extended parallel to suture, not joined to suture near base; abdominal sterna with very fine, transverse microreticulation and a group of shallow punctures at sides; median lobe of aedeagus flattened, blade-like.

##### Description.

*Color*: both individuals are immature and lighter reddish-brown; it is most likely that mature individuals are unicolorous dark brown to piceous, with annexes lighter, reddish-brown. *Microsculpture*: frons, pronotum and elytra with a fine, sometimes barely visible, more or less isodiametric microsculpture, but surface glossy. *Head*: middle part of clypeus more produced than lateral wings, anterior margin slightly concave, bordered, wings unbordered, preocular area finely bordered; upper surface of clypeus glossy without microsculpture, except microscopic fine punctures; divided from frons by a transverse furrow; longitudinal sulci broad, surface microreticulate, bearing a seta at basal level of clypeus on each side; frons glossy, but with fine, sometimes indistinct microreticulation, with a very shallow central foveola; above supraorbital setae with a broad ridge, medially bordering a small longitudinal groove, in this area the microreticulation more distinct; neck finely punctate, interrupted at middle in the holotype, not interrupted in the paratype; posterior margin of eye with the characteristic tubercle. *Prothorax*: convex, disc somewhat flattened, about as wide as long (0.97x, see Table 2); surface glossy, but with fine microreticulation, fine transverse wrinkles in the basal two thirds, declivity at base with transverse wrinkles more dense and more distinctly shagreened; anterior angles markedly produced forward, mediad with a sharp and narrow incision, which corresponds to the postorbital tubercle. *Elytra*: about twice long as wide (range: 1.94× – 1.98×, see Table 2); subparallel, convex, broadest in the apical third, glossy, with very fine, barely visible microreticulation; elytral striae distinctly engraved from base to apex, with gross punctures at base which become finer towards apex; 6^th^ to 8^th^ interval narrowed at base and elevated as a keel; first stria elytral extended more or less parallel to suture, not bent inward toward suture at base; a fine, scutellar keel extended from the basal tubercle at the end of the second interval obliquely posteriorly to suture. *Legs*: fore-tibia 4 dentate, the proximal dentiform projection very small, triangular, upper surface indistinctly sulcate; profemur with a sharp denticle at the proximal end of the outer edge; mesotibia with a small distal spur on the upper edge, which is about two times longer than wide.

**Table 2.** Measurements of *Semiclivina (Uroclivina) schmidi* sp. n.

	**P-LW**	**E-LW**	**L (mm)**	**W (mm)**	**PL (mm)**	**PW (mm)**	**Dl**	**Dr**
**Holotype male**	0.97	1.94	6.17	1.58	1.48	1.53	5.00	5.00
**Paratype female**	0.97	1.98	6.43	1.68	1.56	1.61	5.00	5.00

*Abdominal sterna*: abdominal sterna IV to VII very finely transversally microreticulate, mesh pattern isodiametric at sides, abdominal sternum VII with a group of about 10 fine and shallow punctures on each side, and with 2 pairs of marginal setae in both sexes, relatively close together. *Male genitalia* (Fig. 9 a-c): median lobe blade-like flattened, apex spatulate; parameres long and slender, left one about twice as broad as the right one, with 7 setae at apical margin, right one with a apical fringe of about 10 setae. *Female genitalia, ovipositor*: stylus long, slender, bent inwards, apex acute (Fig. 9 d-e).

**Figure 7.**
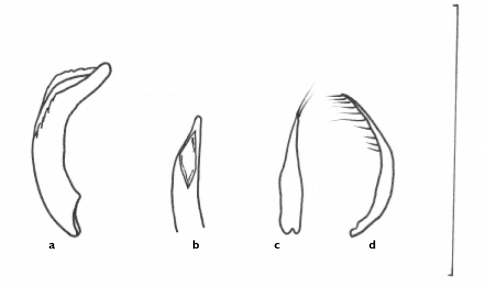
Line drawings of aedeagus of *Semiclivina urophthalmoides* (Kult, 1947), holotype **a** median lobe, left lateral view **b** median lobe, apical portion, dorsal aspect **c** and **d** left and right paramere, respectively, ventral view; Scale bar = 1 mm.

**Figure 8.**
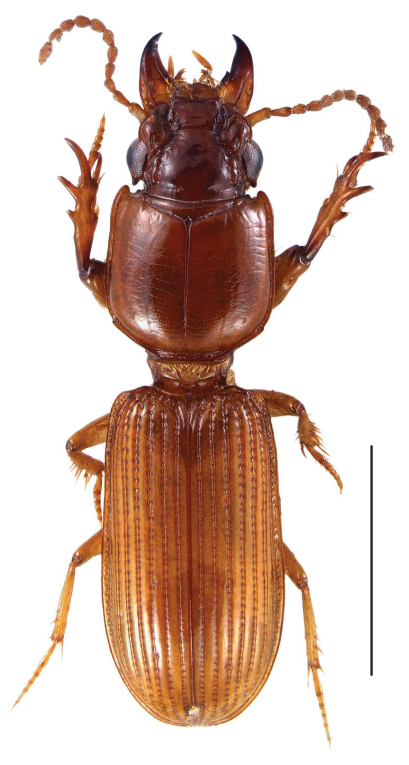
Photograph of habitus of the holotype of *Semiclivina schmidi* sp. n., dorsal view; Scale bar: 2 mm.

**Figure 9.**
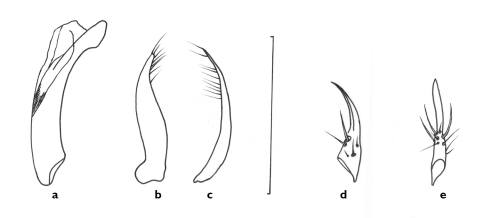
Line drawings of genitalia of *Semiclivina schmidi* sp. n. **a-c** holotype, aedeagus **a** median lobe, left lateral view **b** and **c** left and right paramere, respectively, ventral view **d-e** paratype,ovipositor **d** left stylomere, lateral aspect **e** medial aspect. Scale bar = 1 mm.

##### Measurements.

see Table 2; length 6.17–6.43 mm, width 1.58–1.68 mm.

##### Geographical distribution.

known only from French Guyana

##### Material examined.

*Holotype*: ♂, Fr. Guyana, Mt. Singes, 01.08.2007, leg. Herbert Schmid, (CDW); Paratype: 1 ♀, same dates as Holotype, (CWD).

#### Semiclivina (Uroclivina) urophthalmoides

(Kult, 1947)

http://species-id.net/wiki/Semiclivina_(Uroclivina)_urophthalmoides

Clivina (Semiclivina) urophthalmoides Kult 1947: 34 -35; Van Emden 1949: 861–863; Lorenz 2005: 145; Bousquet 2009: 41

##### Type locality.

Brazil, Mato Grosso, Corumba

##### Holotype.

original series from the same locality, about 30 specimens, holotype in the author’s collection (collection Kult in CDW), paratypes in the collections of ETHZ, IRSNB, MNHN, ZMHB: Kult 1947: 35

##### Geographical distribution.

known from type locality only: Brazil, Matto Grosso, Corumba

##### Material examined.

1 ♂, Holotype with red label “TYPE” and a handwritten determination label “*Semiclivina urophthalmoides* KT.” det. K. Kult 1946, Corumba, Matt. Grosso, (CDW)

##### Annotation.

in the collection of Karel Kult this species is represented by the Holotype (see above for details) and 2 males, 1 female, Corumba, Matt. Grosso, with the labels in red “COTYPE”, without determination labels; 3 males, Corumba, Matt. Grosso, one with the handwritten determination label “*Clivina urophthalmoides* m.” det. K. Kult [without year], most likely also Paratypes of *Semiclivina urophthalmoides* (Kult, 1947), but not indicated as such; 2 Ex., Argentine Republic, Villa Ana, F.C.S. Fe, December 1924, K.J. Hayward, Paratype *Clivina marquardti* Van Emden; 1 female, ditto, January 1926; 1 female, ditto, December 1925, at light; 2 Ex. Paraguay, S. Antonio, with a red printed label “Compared with Type, K. Kult, 1950”. It is noticeable, that the individual labeled as Type is much smaller (4.3 mm) than the other ones. This led to the investigation of the other *Semiclivina urophthalmoides* (Kult, 1947) specimens with dissection of genitalia and abdomen. The result of this investigation was that all other specimens mentioned above except the Holotype, are markedly different (see Figs. 6a-d, 7a-d) and belong to another species, which is described above as *Semiclivina (Uroclivina) bergeri*. The differentiation of these two species is given in the key.

#### Semiclivina (Uroclivina) urophthalma

(Putzeys, 1863)

http://species-id.net/wiki/Semiclivina_(Uroclivina)_urophthalma

Fig. 4d

Clivina urophthalma Putzeys 1863: 37 -38; 1866: 145; Csiki 1927: 512; Van Emden 1949: 862–863 *Clivina (Semiclivina) urophthalma* Putzeys, 1863: Kult 1947: 35; Bousquet 2009: 41

##### Type locality.

Amazonia

##### Holotype.

3 Ex., “Amazone”: Putzeys 1863: 38; Coll. Putzeys (IRSNB).

##### Geographical distribution.

Amazonia

##### Material examined.

1 Ex Brazil: Para, (CDW).

##### Annotation.

Kult 1947: 35 examined the type of *Clivina urophthalma* Putzeys, 1863 and compared it with his *Clivina urophthalmoides* Kult, 1947; Van Emden 1947: 862–863 did this as well, both authors agreed in the interpretation of *Clivina urophthalma* Putzeys, 1863 in comparison to *Clivina urophthalmoides* Kult, 1947. The specimen of *Clivina urophthalma* Putzeys, 1863 in Kult’s collection fits very well Putzeys’ description (Putzeys 1863: 37–38) and the interpretation of above mentioned authors.

#### Semiclivina (Uroclivina) oxyomma

(Putzeys, 1868)

http://species-id.net/wiki/Semiclivina_(Uroclivina)_oxyomma

Fig. 4c

Clivina oxyomma Putzeys 1868: 10; Csiki 1927: 509; Van Emden 1949: 862–863
Clivina (Semiclivina) oxyomma  Putzeys, 1868: Kult 1947: 35, Bousquet 2009: 41

##### Type locality.

Brazil: Bahia

##### Holotype.

1 Ex. Coll de Castelnau (MNHN): Putzeys 1868: 10

##### Geographical distribution.

Brazil: Bahia

##### Material examined.

1 ♀, Brazil: Tapajos, (CDW).

##### Annotation:

Van Emden 1947: 862–863 examined type material of *Clivina oxyomma* Putzeys, 1868, the specimen represented in Kult’s collection fits well Van Emden’s interpretation of this species and the short description of Putzeys (1868: 10).

## Supplementary Material

XML Treatment for Semiclivina

XML Treatment for Uroclivina

XML Treatment for Semiclivina (Uroclivina) bergeri

XML Treatment for Semiclivina (Uroclivina) schmidi

XML Treatment for Semiclivina (Uroclivina) urophthalmoides

XML Treatment for Semiclivina (Uroclivina) urophthalma

XML Treatment for Semiclivina (Uroclivina) oxyomma
